# γ-Tocotrienol and 6-Gingerol in Combination Synergistically Induce Cytotoxicity and Apoptosis in HT-29 and SW837 Human Colorectal Cancer Cells

**DOI:** 10.3390/molecules200610280

**Published:** 2015-06-03

**Authors:** Khairunnisa’ Md Yusof, Suzana Makpol, Rahman Jamal, Roslan Harun, Norfilza Mokhtar, Wan Zurinah Wan Ngah

**Affiliations:** 1 UKM Medical Molecular Biology Institute (UMBI), UKM Medical Center, Jalan Ya’acob Latiff, Bandar Tun Razak, Cheras 56000, Malaysia; E-Mails: rahmanj@ppukm.ukm.edu.my (R.J.); drroslan@ppukm.ukm.my (R.H.); norfilza@yahoo.co.uk (N.M.); 2 Department of Biochemistry, Faculty of Medicine, Universiti Kebangsaan Malaysia, Jalan Ya’acob Latiff, Bandar Tun Razak, Cheras 56000, Malaysia; E-Mail: suzanamakpol@yahoo.com

**Keywords:** γ-tocotrienol (γ-T3), 6-gingerol (6G), synergistic, HT-29, SW837 human colorectal cancer cells

## Abstract

Numerous bioactive compounds have cytotoxic properties towards cancer cells. However, most studies have used single compounds when bioactives may target different pathways and exert greater cytotoxic effects when used in combination. Therefore, the objective of this study was to determine the anti-proliferative effect of γ-tocotrienol (γ-T3) and 6-gingerol (6G) in combination by evaluating apoptosis and active caspase-3 in HT-29 and SW837 colorectal cancer cells. MTS assays were performed to determine the anti-proliferative and cytotoxicity effect of γ-T3 (0–150 µg/mL) and 6G (0–300 µg/mL) on the cells. The half maximal inhibitory concentration (IC_50_) value of 6G+ γ-T3 for HT-29 was 105 + 67 µg/mL and for SW837 it was 70 + 20 µg/mL. Apoptosis, active caspase-3 and annexin V FITC assays were performed after 24 h of treatment using flow cytometry. These bioactives in combination showed synergistic effect on HT-29 (CI: 0.89 ± 0.02,) and SW837 (CI: 0.79 ± 0.10) apoptosis was increased by 21.2% in HT-29 and 55.4% in SW837 (*p* < 0.05) after 24 h treatment, while normal hepatic WRL-68 cells were unaffected. Increased apoptosis by the combined treatments was also observed morphologically, with effects like cell shrinkage and pyknosis. In conclusion, although further studies need to be done, γ-T3 and 6G when used in combination act synergistically increasing cytotoxicity and apoptosis in cancer cells.

## 1. Introduction

The search for chemopreventive compounds of plant origin for the development of drugs or adjuvants in the treatment of cancer. Continues mounting evidence suggests that bioactives present in plants have a significant impact in reducing the recurrence of cancer [1,2]. Examples of bioactive compounds that possess anti-cancer properties shown by biomedical studies include epigallocatechin gallate, resveratrol, genistein, quercetin, sulforophane and curcumin [2,3]. For example, curcumin has been shown to sensitize tumor cells and act in first line chemotherapy and radiotheraphy due to its bioavailability in the human body and it exerts its therapeutic effect at minimum concentrations in blood serum [4]. The success of curcumin, which is now being tested in clinical trials, has spurred research into other plant bioactives.

6-Gingerol (6G) is a major bioactive of ginger and has been reported to have anti-cancer and antioxidant properties [5]. The anti-cancer potential of 6G has been reported in both *in vivo* and *in vitro* studies. Previous findings have demonstrated that 6G treatment in colorectal cancer cells caused mitochondrial damage and inhibited cell survival pathways [6].

Vitamin E exists in different isoforms such as tocotrienol and tocopherol that have been shown to have anti-cancer properties. Tocotrienol displayed potent antiproliferative and apoptotic activity against mammary tumor cells at concentrations that have no adverse effect on normal cell growth or viability *in vitro* [7]. Furthermore, the isoforms of tocotrienol may have different biological activities where δ-tocotrienol is more potent as an anti-proliferative agent in prostate cancer cells, followed by γ-tocotrienol, β-tocotrienol and α-tocotrienol [8], but in HeLa cells, γ-tocotrienol (γ-T3) is more potent compared to δ-tocotrienol [9]. Tocotrienol also induced apoptosis in human gastric carcinoma SGC-7901 and human colon carcinoma HT-29 cells, and has been associated with suppression of the Raf-ERK signalling pathway [10], mitogen-activated protein kinase signalling pathway [11], and inhibitory effects on cell invasion and metastasis [12].

Most of the reported studies on inhibitory effects of bioactive compounds involved the use of chemo-preventive agents which have limited bioavailability while higher doses can sometimes lead to increased toxicity. The use of a combination of low concentrations of preventive agents, or multi targeted approaches has been suggested to reduce toxicity and improve efficacy of the treatment [13,14,15,16]. In theory, a combination of chemopreventive agents also permits administration of lower concentrations of each compound thereby minimizing the risk of adverse effects [13] and overcoming bioavailability issues. However, studies using bioactives in combination are very limited. Considering the heterogeneous nature of cancer cells, testing of bioactives may require the use of several types of cancer cell lines. Cancer cell lines of different stages may also vary in their response to treatment. Thus, the objective of the present study was to determine the effect of γ-tocotrienol and 6-gingerol individually and in combination on human colorectal cancer cells.

## 2. Results and Discussion

### 2.1. Effect of Individual 6G and γT3 and in Combination on Cell Viability 

**Figure 1:**
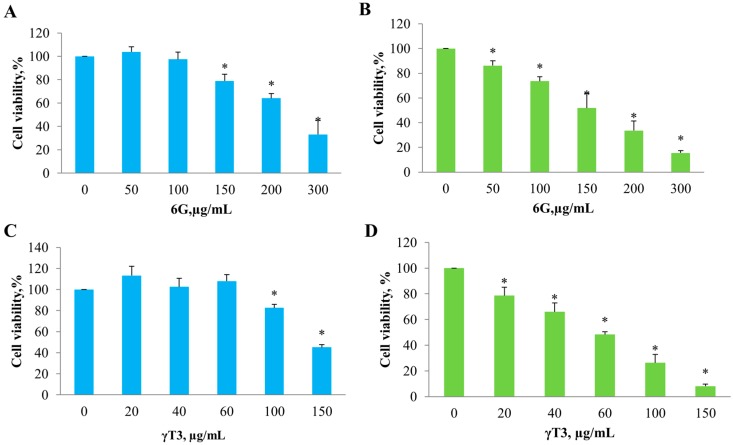
Cell viability assay of individual 6G and γ-T3 of both untreated and treated HT 29 (**A**,**C**) and SW 837 (**B**,**D**) cells after 24 h treatment. HT 29 and SW 837 cells were treated with 6G (0, 50, 100, 150, 200, and 300 µg/mL) and γ-T3 (0, 20, 40, 60,100, 150 µg/mL). Cell viability was observed using ELISA microplate reader at 405nm. Values are expressed as mean ± SEM from three independent experiments in triplicates (*n* = 3). * significant when compared with untreated cells (*p* < 0.05).

MTS assays of individual 6G and γ-T3 were carried out on both HT-29 and SW837 cells at concentrations ranging from 0 to 300 µg/mL for 6G and 0 to 150 µg/mL for γ-T3. Both compounds caused a concentration-dependent decrease in cell viability in HT-29 and SW837 cells (Figure 1). IC_50_ values obtained for 6G on HT-29 was 254.0 ± 42.0 and 158.4 ± 20.5 for SW837, while they were 138.9 ± 9 and 57.7 ± 5.8 µg/mL for HT-29 and SW837 after treatment with γ-T3 (Table 1).

**Table 1:** MTS assay results for individual 6-gingerol and γ-tocotrienol treatments on each cell line. Data are expressed as mean ± SD, in three independent experiments (*n* = 3).

Cell Lines	Bioactive Compound	IC_50_ Value (µg/mL)	* Cell Viability, %
**HT-29**	6-Gingerol (6G)	254.0 ± 42.0	40.1 ± 18.0
	γ-Tocotrienol (γ-T3)	138.9 ± 8.7	41.1 ± 8.4
**SW837**	6-Gingerol (6G)	158.4 ± 20.5	13.4 ± 2.8
	γ-Tocotrienol (γ-T3)	57.7 ± 5.8	8.0 ± 1.9

* Percentage of cell viability after 24 h treatment at maximum concentration, 300 µg/mL for 6-gingerol, and 150 µg/mL for γ-tocotrienol.

**Figure 2:**
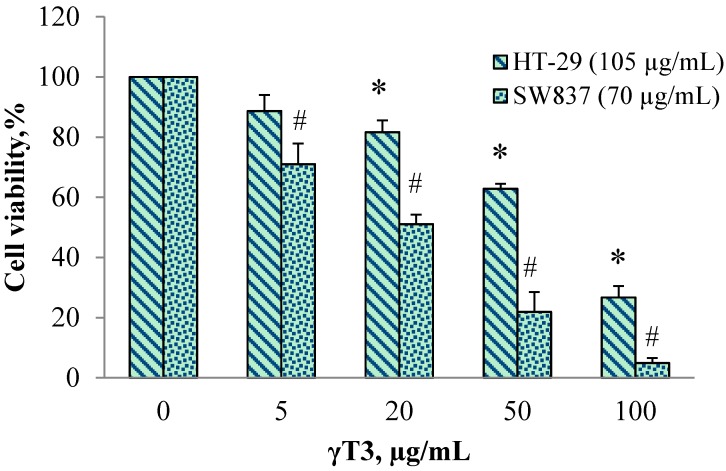
Cell viability assay of combined 6G and γ-T3 on cells after 24 h treatment. HT 29 cells were treated with 105 µg/mL 6G while SW 837 treated with 70 µg/mL 6G in combinations of γ-T3 (0, 5, 20, 50, and 100 µg/mL). Cell viability was measured using ELISA microplate reader at 405 nm. Values are expressed as mean ± SEM from three independent experiments (*n* = 3). * Compared with untreated HT-29 cells (*p* < 0.05), # compared with untreated SW 837 cells (*p* < 0.05).

**Figure 3:**
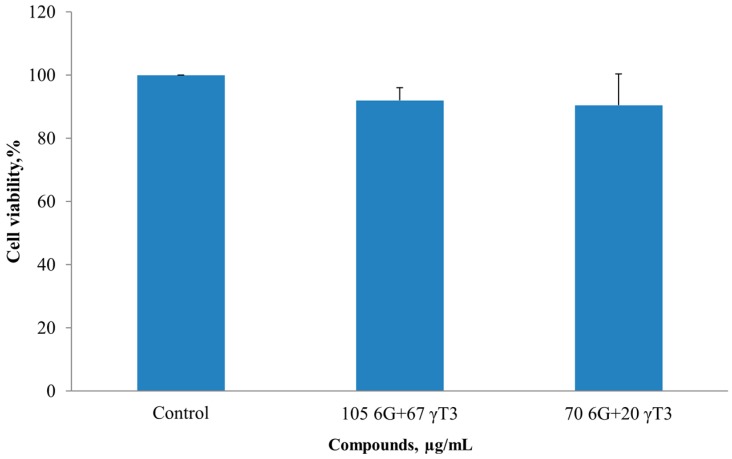
Combined treatment, 6G+γ-T3 after 24 h had no effect on human hepatic cells, WRL-68. No significance difference was observed between treated and untreated cells. Values are expressed as mean ± SEM from three independent experiments (*n* = 3).

Subsequent cell viability tests were done by using sub-half maximal individual 6G concentrations, which was 105 for HT-29 and 70 µg/mL for SW837, in combination with γ-T3 at varying doses (0, 5, 20, 50 and 100 µg/mL). The new IC_50_ values obtained for 6G+γ-T3 combined were 105 + 67 µg/mL and 70 + 20 µg/mL for HT 29 and SW 837 cells, respectively. The combination index was also calculated (Table 2). The combination treatment showed inhibitory effects in a concentration-dependent manner (Figure 2). Normal hepatic WRL-68 cells were unaffected when treated with the IC_50_ concentration of 6G+γT3 obtained from both HT-29 and SW837 results (Figure 3).

**Table 2:** Cell viability, IC_50_ value, and combination index for combined 6G+γ-T3 on each cell lines. Data are expressed as mean ± SD, of three dependent experiments (*n* = 3).

Cell Lines		γT3 (µg/mL)					Combination Index, C.I
	0	5	20	50	100	IC_50_ Value	
**HT-29 (105 µg/mL 6G)**	100	88.6 ± 9.2	81.6 ± 6.9	62.8 ± 8	26.7 ±6.5	67.0 ± 3.0	0.89
**SW837 (70 µg/mL 6G)**	100	70.9 ± 9	51.1 ± 9.7	21.9 ± 10.9	4.9 ± 3.9	20.1 ± 8.8	0.79

### 2.2. Isobologram Analysis of 6G+γT3 of HT-29 and SW837 Cells

**Figure 4:**
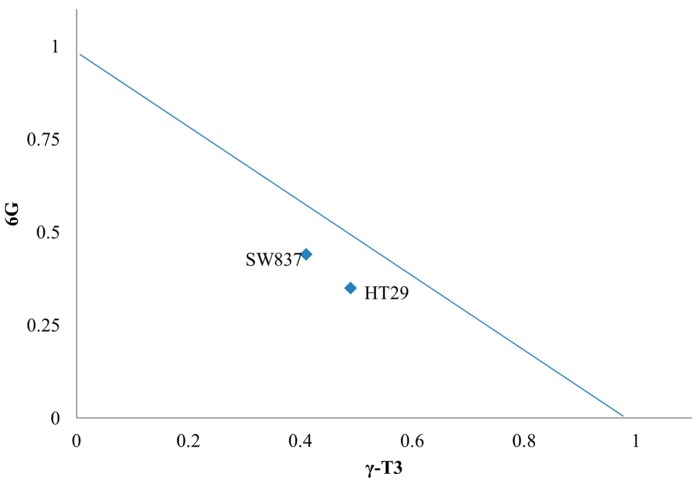
Isobolographic analysis of combined treatment of 6G with γ-T3 on both HT-29 (CI = 0.89) and SW837 (CI = 0.79). The line represents as a line of additivity. Values below the line indicates synergistic interaction (CI < 1), while above is considered as antagonistic interaction (CI > 1) with values on the line indicating additive interaction (CI = 1).

The aim of this study is to determine if the combination of 6G and γ-T3 induces a synergistic interaction at low concentrations to cause cell death. Thus, an isobologram was used to calculate the interaction effect of 6G and γ-T3 on both cell lines. Isobolographic analysis showed that the combination of both bioactives induced a synergistic interaction in both HT-29 and SW837 after 24 h treatment (Figure 4)

### 2.3. Morphological Changes of Combined Treatment 6G with γ-T3 on HT-29 and SW837 Cells

**Figure 5:**
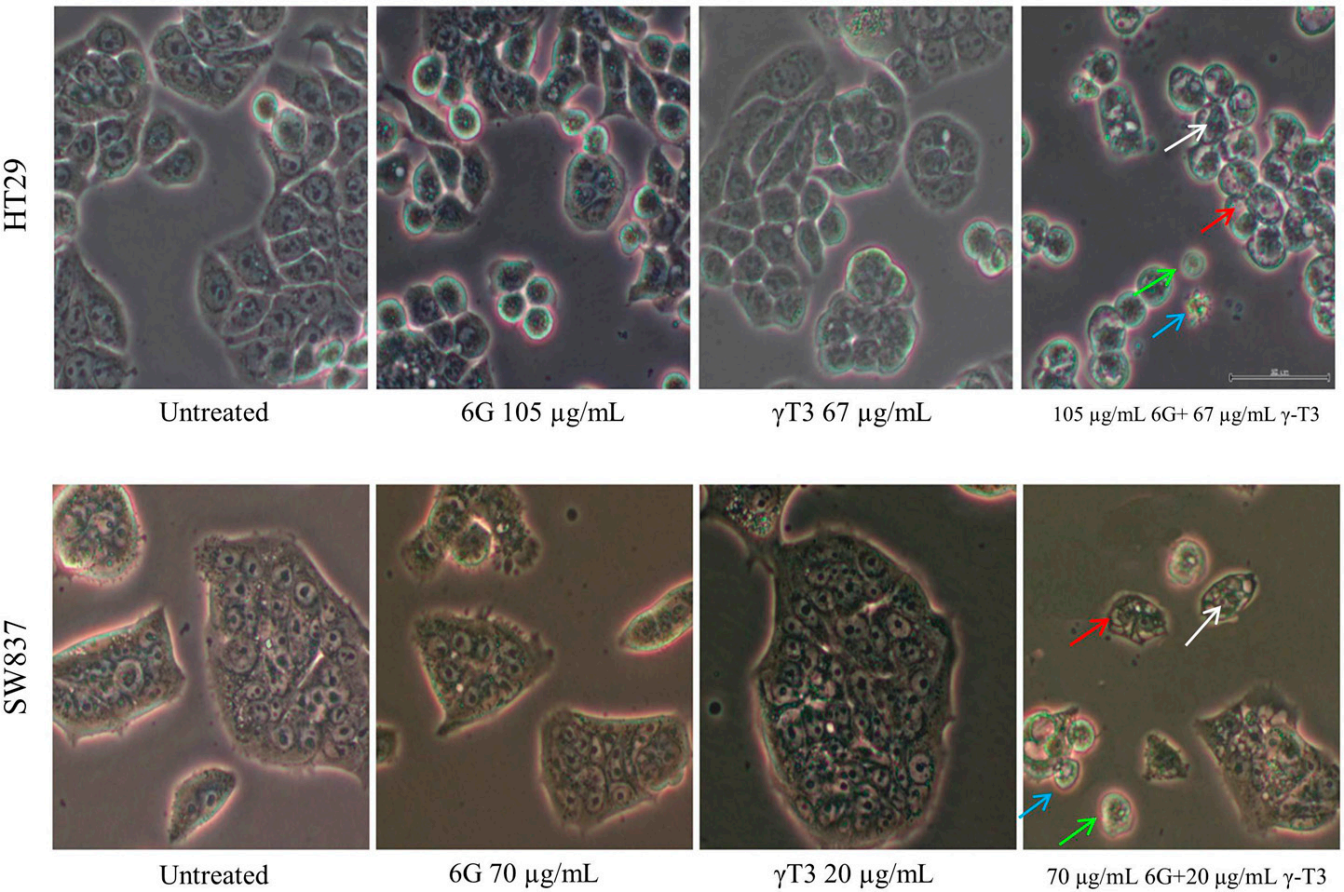
Phase-contrast microscopic observation of untreated HT-29 and SW837 cells displayed intact, strong attachment with adjacent cells as well as prominent appearance of cytoplasm and nucleus. Cells that were treated with 6G and γ-T3 alone also displayed intact cells, similar to untreated cells. Dramatic changes in cell morphology were observed with combined treatment of 6G and γ-T3 after 24 h. Cells were reduced in number, became rounded and distorted, lost contact with adjacent cells, and there were more floating cells (**green arrow**), pyknosis (**white arrow**), and apoptotic bodies (**blue arrow**). Furthermore, appearance of vacuoles in the cytoplasm of treated cells was also observed (**red arrow**) (40×).

The induction of cell death via apoptosis by combined 6G and γ-T3 treatment was observed using a phase contrast microscope to determine the morphological changes in untreated and treated cells. The cells of control, and sub-lethal concentration of individual 6G and γ-T3 treatment remained unchanged while 6G+γ-T3 treatment displayed distortion and shrinkage of cells, indicating cell death (Figure 5).

### 2.4. Effects of Combined 6G+γ-T3 on Apoptosis of HT-29 and SW837 Cells

**Figure 6:**
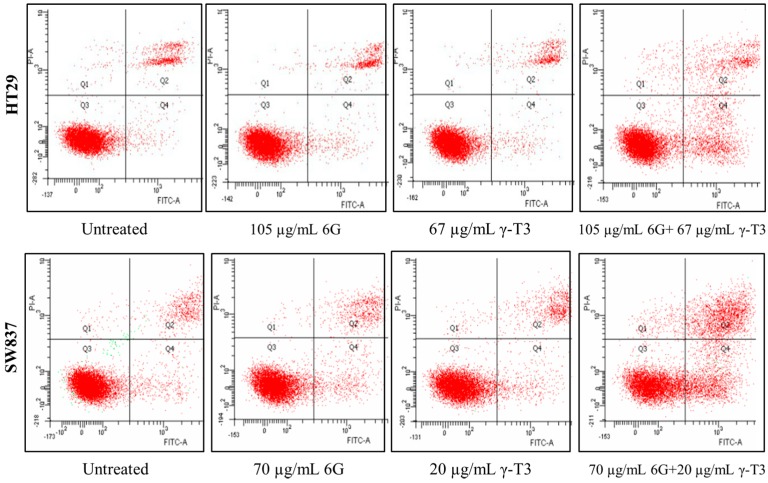
Effects of 6G, γ-T3 and 6G+γ-T3 on both HT 29 and SW 837 cells. Apoptosis was quantified using flow cytometry. Q1 represents dead cells/necrosis, An−/PI+. Q2 late apoptosis, An+/PI+, Q3 viable cells An−/PI−, and Q4 early apoptosis, An+/PI−.

**Figure 7:**
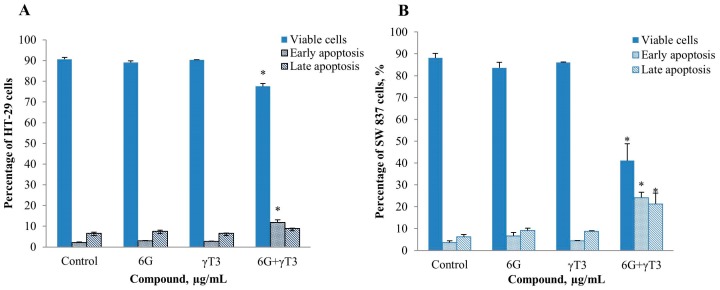
Annexin V FITC analysis on control, sub-lethal concentration of individual 6G and γ-T3, and combined 6G+γ-T3 treatment on both (**A**) HT-29 and (**B**) SW837 cells. The number of cells decreased (*p* < 0.05) in 6G+γ-T3 treatment as compared to control, both in HT-29 and SW837 cells. No significant difference was observed between individual treatment of 6G and γ-T3 when compared to control cells. Percentage of viable, early and late apoptotic cells. Data are presented as mean ± SEM of three independent experiments. * *p* < 0.05 *vs.* control.

**Figure 8:**
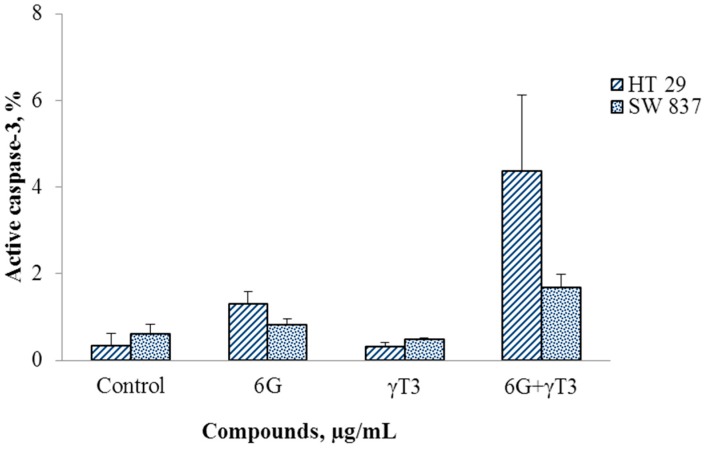
Combined of 6G+γ-T3 treatment did not increase active caspase-3 in both HT-29 and SW837. There was also no significant difference of active caspase-3 determined in sub-lethal concentrations of individual 6G and γ-T3 treatments. Data are presented as mean ± SEM of three independent experiments.

The IC_50_ values of combined 6G+γ-T3 treatment of cells were used to determine the mode of cell death which was assayed by flow cytometry using annexin V FITC and active caspase-3 staining. Cytogram analysis suggested that the cells undergo apoptosis as the cells died in early and late apoptosis (Figure 6). No significant difference was observed between control and sub-lethal concentration of individual 6G and γ-T3 treatment but combined treatment of 6G+γT3 increased cell death (*p* < 0.05) (Figure 7). Active caspase-3 is a key protease activated during early apoptosis, both in intrinsic and extrinsic pathway. In this study, active caspase-3 was not increased in all treatments after 24 h for both cells (Figure 8).

Interest has increased in bioactive compounds present in plants which can modulate the development and progression of cancer. Accumulating evidence has suggested that many cancers are preventable, an effect attributed to dietary constituents, particularly phytochemicals which can modulate multiple stages carcinogenesis as well as reduce cancer risks [17]. Promising anti-cancer agents including bioactive compounds such as curcumin in turmeric [18] and epigallocatechin gallate (EGCG) found in green tea [19] have reached clinical trials and shown promising results that can be further investigated for use as therapeutic agents. Currently, hundreds of bioactive compounds are being studied experimentally and clinically as chemopreventive agents.

Gingerol, a major constituent of ginger was found to have antioxidant [20], anti-inflammatory [21], anti-genotoxic [22], anti-arthritic [23] and anti-cancer effects [5,24,25]. In this study, 6G alone was found to be effective in inhibiting growth of HT-29 and SW837 cells in a concentration dependent manner. This finding was consistent with a previous study that showed anti-proliferative effect of 6G in HPAC and BxPC-3 pancreatic cells [26], HeLa cells [5] and PC-3 prostate cancer cells [27].

In our study, the IC_50_ values of 6G on HT-29 and SW837 were 254 µg/mL and 158 µg/mL, respectively, after 24 h treatment. The range of concentrations was consistent with the previous study using HeLa cells where the IC_50_ of 6G after 24 h treatment was 126 µg/mL [6]. Other recent studies reported 6G reduced 50% of SW480 colon cancer cells growth at a concentration of 60 µg/mL or 205 ± 5 µM [28] and 28% of HT-29 and HCT116 at concentration of 200 µM after 72 h treatment [6]. The lower concentration of 6G needed could be due to the longer treatment time and a different method used in determining cell cytotoxicity. We believe that the concentration of 6G used in this study is achievable as daily human consumption of approximately 250 mg–1 g of ginger contains up to 1.0%–3.0% of 6G [29]. In addition, 6-gingerol present in ginger has a high distribution in tissues of the gastrointestinal tract due to its lipophilicity [30] with a short half-life of 75 to 120 min in human plasma [31]. The other compound used in this study which was γ-T3 which is an isoform of vitamin E that has been suggested to have antioxidant properties and anti-cancer effects on a wide range of cancer cells including human colon carcinoma [32,33,34], prostate [35,36], mammary [37,38], stomach [11,15], liver [39] and skin cancer [40]. In our study, γ-T3 significantly inhibited cell growth of both HT-29 and SW837 cells after 24 h treatment, in a concentration dependent fashion. The IC_50_ of γ-T3 for HT-29 and SW837 was 138 µg/mL (equivalent to 336 µM) and 58 µg/mL (141 µM). This concentration was higher than that reported in other studies using DU145 prostate and MGH-U1 bladder cancer cells with IC_50_ values of 45 to 60 µg/mL [36], 31.4 ± 1.51 µM using SW 620 colon cancer cells [34] and less than 50 µM using MCF-7 and MDA-MB-231 human mammary cells [38]. The differences observed could be due to different number of cells and different incubation times used.

The inhibition of cell growth by 6G and γ-T3 alone on various cancer cells was reported in previous studies suggesting that anti-proliferative effect of these compounds on cancer cells is consistent. According to Lee *et al.* [6], 6G inhibits cell growth of HCT116, HT-29, SW480, LoVo and Caco-2 colorectal cancer cells by multiple mechanisms, such as by inhibiting transcription of cyclin D1 through the suppression of β-catenin, down-regulating cyclin D1 resulting in cell growth arrest and up-regulating NAG-1 expression through the GSK-3β and PKCε pathways. Both cell lines used were *APC*-mutant cells. It has been known that colon cancer with a mutant *APC* gene contained high levels of free β-catenin [41]. 6G has been reported to suppress β-catenin in colon cancer cells through β-catenin/TCF-dependent gene transcription to induce anti-tumorigenesis effect shown by the reduction of cyclin D1 resulting in G1/S phase arrest as well as decreased β-catenin localization in cells [6]. The similarities between HT-29 and SW837 cell lines were not only in *APC* mutation, but also in the *p53* mutated gene. 6G is reported to modulate *p53* levels in HCT-116 and LoVo cells which are *p53*-wild type cells, but not in SW-480, a *p53*-mutant cell [6,42]. The results observed are consistent with the results for BxPC-3 mutant *p53* pancreatic cancer cells where 6G treatment (400 µM) showed a decline in p53 level but overexpression of p21 suggesting that cell death might be caused by *p53*-independent events [26]. It has been reported that inactivation of *p53* results in chemoresistance by chemopreventive agents [6]. Mutation in *p53* decreased sensitivity to G1/S interface of cell cycle in prostate cancer cells [43]. Thus, it is possible that 6G effects on cell cycle may depend on *p53* status.

Previous studies have shown that γ-T3 suppresses cell growth by reducing cyclin D1, CDK4, CDK6, and CDK2 levels in mammary cancer cells [44] and SW620 human colon carcinoma [34]. Cyclin D1, a protein that forms a complex with CDK4/6, mediates a growth factor involved in G1 phase progression. Thus, a decrease in cyclin D1 results in G0/G1 phase arrest [9,26]. Like 6-gingerol, γ-T3 also suppressed transcriptional activity of β-catenin/Tcf signalling in both *APC*-mutant cells of HT-29 cells [33], SW620 human colon carcinoma [34] and in *APC*-wild type prostate cancer stem cells [36]. It is also reported γ-T3 is associated with the inhibition of the NF-kappa B (NF-κB) signalling pathway, a cell survival regulator which is frequently up-regulated in cancer. Inactivation of NF-κB by γT3 was reported in PCa prostate cancer cells [35], Taken together, both 6G and γ-T3 may exert their anti-proliferative effect in HT-29 and SW837 which are *APC* and *p53*-mutant cells by modulating cyclin D1 in β-catenin/Tcf signalling pathway which later causes cell cycle arrest.

The idea of combining two or more bioactives as treatment may be helpful in reducing the toxicity and adverse effects of cancer treatment. Synergistic interactions have favourable outcomes such as enhanced efficacy, the need for lower concentrations or dosages at equal or increased level of efficacy, and simultaneous enhancement of therapeutic actions as well as reduction of unwanted actions during treatment [45,46,47]. It is noted that anti-cancer therapy is more effective when multiple drugs with complimentary mechanisms and molecular targets are given to optimize the synergistic therapeutic response [7]. Synergistic interactions were identified between combinations of indole-3-carbinol and genistein to reduce the growth and induce apoptosis of human colon cancer HT-29 cells [48], while concurrent treatment of sulforaphane and eugenol synergistically induces cell cytotoxicity and apoptosis [49] and co-treatment of sesamin with γ-tocotrienol inhibited the proliferation and caused cytostatic effects in mouse and human mammary cancer cells [50].

In this study, we used sub-half maximal inhibitory concentrations of 6G alone, 105 µg/mL and 70 µg/mL on HT-29 and SW 837 cells with different γ-T3 concentrations. The inhibitory effect of 6G+γ-T3 in both HT-29 and SW837 cells occurred in a concentration dependent manner. The lower IC_50_ of the combined treatment obtained for HT-29 and SW837 suggested a synergistic interaction based on the isobologram index (CI < 1, 0.89 for HT-29 and 0.79 for SW837). The respective concentrations (IC_50_) had no effect on WRL-68 human hepatic cells after 24 h of treatment, consistent with a previous study that reported that γ-T3 showed no cytotoxicity on normal human peripheral blood mononuclear cells [51]. Normal intestinal epithelial cells were unaffected when treated with high concentrations of 6G (500 µM) [28] and human fibroblast cells were only inhibited at 500 µM [52].

A number of studies have shown that combined treatment of γ-T3 with other chemotherapeutic agents induced synergistic interaction to enhance cytotoxic or cytostatic effects on tumor cells. γ-T3 in combination with resveratrol has been reported to enhance the apoptosis effects in estrogen receptor positive MCF-7 breast cancer cells [53]. Combined treatment of sesamin and γ-T3 also showed an synergistic interaction and inhibited the growth of murine +SA, human MCF-7 and human MDA-MB231 mammary tumor cells [50]. Other studies of γ-T3 combined with specific drugs such as statin [54], celecoxib [55], and erlonitib/gefitinib [56] displayed significant chemopreventive effects against various cancers, as compared to individual treatments alone. In other studies, it was reported that treatment with γ-T3 alone significantly reduced quinone reductase, NQO1 while EGCG markedly increased the enzyme but when both compounds worked concurrently, they elicit synergism by boosting the NQO1 production in MCF-7 breast cancer cells [53]. Since both 6G and γ-T3 have been reported to produce a variety of biological effects, we hypothesized that multiple pathways were probably involved. 6G exerts its anti-inflammatory properties by affecting various pro-inflammatory factors such as TNF, COX2 and PGE_2_ [57,58] while γ-T3 alone may act as an angiogenic inhibitor by modulating the PI3K/PDK/Akt signalling pathway [59] and down-regulating VEGF and VEGF receptors in the ERK-signaling pathway in SGC-7901 gastric adenocarcinoma cells [10]. Perhaps, the different molecular targets affected by 6G and γ-T3 accelerate the cytotoxic and cytostatic effects. Furthermore, a previous study on different prostate cancer cell lines, namely DU145, LNCaP, and PC3 showed that γ-T3 exerts its anti-cancer action by modulating different mechanism routes as it depends on the existence of androgen receptors on cells [60]. In the present study, as HT-29 carried *BRAF*, *SMAD4* and *PI3KCA*-mutated genes while SW837 is a *KRAS* mutated cell, hence different mechanisms may contribute to increased cell death.

The inhibitory effect of the combined treatment is accompanied by apoptosis, shown by morphological changes of the cells. We observed significant characteristics of apoptotic cells in both 6G+γT3 treated cells, such as cell shrinkage, pyknosis (chromatin condensation), rounded and distorted cells, reduced adherence to adjacent cells, and blebbing of plasma membrane (Figure 5). Furthermore, the apoptosis effect of 6G+γ-T3 is further supported by significant externalization of phosphotidylserine suggesting early apoptosis and existence of apoptotic bodies in late apoptosis (Figure 6). Later, cells disintegrate into secondary necrosis, another programmed cell death form which is distinguished from necrosis without scavengers such as macrophages to remove the apoptotic bodies before proceeding to autolytic necrotic outcomes [61,62,63]. As both HT-29 and SW837 cells died following the apoptosis sequence, it can be suggested that the cells died through the apoptotic pathway rather than due to necrosis. We also observed a difference in sensitivity of the two types of cells towards 6G+γ-T3 treatment, as HT-29 cells were more resistant compared to SW837 in cell viability and apoptosis. A previous study showed that SW-620 clones expressing *SMAD4* were three fold more sensitive to 5-FU treatment when compared with control based on the IC_50_ values. Besides, treatment on SW-620 cells that were *SMAD4-*deficient with 5-FU increased apoptotic cells by 26.3% ± 3.4% compared to 47.3% ± 6.2% in *SMAD4* expressing clone cells [64]. HT-29 carries the *SMAD4* mutation, a tumor suppressor gene located at 18q21 chromosome. It has been reported that deficiency of *SMAD4* exhibits tumor progression, up-regulates VEGF expression related to vascular density progression, and chemoresistance to 5′-fluorouracil, anti-cancer drug-mediated apoptosis [65,66] and patients with high levels of *SMAD4* have better survival rates than patients with low *SMAD4* levels in CRC [67,68]. Moreover, loss of *SMAD4* in CRC plays an important role in chemoresistance to 5-FU by activating the PI3K/Akt pathway and regulating cell cycle and apoptosis-related proteins [68]. In CRC that are *SMAD4* deficient, the changes of c-Myc and cyclin D1 increased levels and down-regulation of p21 and p27 are associated with a decrease of phosphorylation of Rb which later results in cell proliferation. Following the inhibition of *SMAD4*, the Akt pathway is activated and the level of anti-apoptotic proteins such as Bcl-2 and survivin increased, and down-regulates pro-apoptotic keys, Bad and Bim, and finally suppressed apoptotic effects in cancer cells [68,69]. As both 6G and γ-T3 were reported to have effects on the PI3K/Akt signalling pathways, we hypothesized that the resistance of HT-29 towards 6G+γ-T3 treatment could be due to dysregulation of *SMAD4* through the PI3K/Akt signalling pathway.

By using morphological changes and membrane alteration evidence, we further tested the apoptotic effect of 6G+γ-T3 by activation of caspases via detection of active caspase-3. After 24 h of 6G+γ-T3 treatment, no significant production of active caspase-3 was observed in treated cells. Paradoxically, both 6G and γ-T3 alone were reported to mediate apoptosis in various cancer cell lines via the caspase-3 cascade, regardless by intrinsic or extrinsic pathways [6,28,36,56,57]. In this study, neither compound alone produced significant active caspase-3 after 24 h. A study done by Kim *et al.* [60] reported that production of caspase-3 after 100–300 µM of 6G treatment of LNCaP prostate cancer cells was not evident at 24 h, but significant expression was observed after 48 h. In another study, γ-T3 and δT3 did not show significant activation of caspase-8 and caspase-3 in DU145 and PC3 prostate carcinoma cells at IC_50_ concentrations, suggesting that tocotrienols could also mediate apoptosis by caspase-independent programmed cell death [70]. The findings were similar to a study done by Takashi and Loo [71] who reported that PARP, an enzyme involved in DNA repair which can be cleaved by caspase-3 during apoptosis was undetectable in MDA-MB-231 human breast cancer cells after treatment with γ-T3.

It is interesting to note that our findings showed apoptosis was increased without caspase-3 activation in 6G and γ-T3 alone as well as in combination treatment in both cell lines. The other possible explanation of this circumstance is that 6G+γ-T3 induced apoptosis synergistically by activating caspase-independent pathways as well. It has been reported that more than one type of programmed cell death may be activated at the same time [72]. Therefore further studies are required to elucidate the mode of action of these bioactives in inducing apoptosis.

## 3. Experimental Section

### 3.1. Cell Culture

Human colorectal cancer HT-29 and SW837 were obtained from ATCC (Manassas, VA, USA). HT-29 cells were maintained in McCoy’s medium (Gibco, Paisley, Scotland) in 25 cm^2^ vented flasks at 37 °C in a 5% CO_2_ atmosphere at constant humidity while SW837 cells were maintained in Leibovitz L-15 medium (Gibco) in 25 cm^2^ non-vented flasks at 37 °C with no CO_2_ atmosphere humidity. Both media were supplemented with 10% (*v*/*v*) heat-inactivated fetal bovine (PAA, Piscataway, NJ, USA), 1% antibiotic penicillin/streptomycin (PAA) and 1% antifungal amphotericin B (PAA). Medium was changed 2–3 times per week. For subculturing, cells were rinsed with phosphate buffered saline and incubated with Accutase (PAA) approximately 5 min for cell detachment. All tests and assays were done after the cells achieved 80%–90% confluence. The final ethanol concentration in all tests was maintained under 0.5%.

### 3.2. Chemicals and Materials

The bioactive compound 6-gingerol (≥ 98% HPLC purity) was obtained from Sigma Aldrich (St. Louis, MO, USA) while γ-tocotrienol (γ-T3, ≥ 97% purity) was purchased from Davos Life (Singapore). FITC Active Caspase-3 Apoptosis Kit (BD Bioscience, Franklin Lakes, NJ, USA), FITC Annexin V Apoptosis Detection Kit II (BD Bioscience), (3-(4,5-dimethylthiazol-2-yl)-5-(3-carboxymethoxyphenyl)-2-(4-sulfophenyl)-2*H*-tetrazolium, inner salt) or MTS (Promega, Madison, WI, USA) were also used.

### 3.3. Cell Viability Assay

The effect of 6G and γ-T3 on cell viability was determined by the MTS assay. Briefly, cells were seeded in 96-well plate (BD Biosciences, Franklin Lakes, NJ, USA), at 2 × 10^4^ of HT-29 cells and 1 × 10^4^ of SW837 cells per well. Concentrations used for 6G and γ-T3 were 0, 50, 100, 200 and 300 µg/mL and 0, 20, 40, 60, 100 and 150 µg/mL respectively. After 24 h of incubation, medium was removed and 20 µL of (3-(4,5-dimethylthiazol-2-yl)-5-(3-carboxymethoxyphenyl)-2-(4-sulfophenyl)-2*H*-tetrazolium, inner salt) or MTS solution and 100 μL medium were added. The plate was incubated for 2 h before reading using an ELISA microplate reader at 405 nm wavelength. Effect of growth inhibition by treatment was calculated as percentage of viability, using vehicle only as 100%. For combination treatment, MTS assay was done to obtain the IC_50_ of the combination by using half of IC_50_ of 6G and combined with titrated γ-T3 at concentrations of 0, 5, 20, 50, 100 µg/mL. The new IC_50_ values were obtained and subsequently used in further tests. Concentrations of combination of both HT-29 and SW837 were also tested on human normal hepatic cell, WRL-68 at the density of 1 × 10^4^ and 2 × 10^4^ cells in each well.

### 3.4. Isobologram and Combination Index Analysis

An isobologram was used to calculate the combination index (CI). A CI value < 1, = 1, and > 1 are considered as synergistic, additive, and antagonistic interaction respectively [73]. The combination index is calculated as shown below:

Combination index (CI) = d_x_/D_X_ + d_y_/D_Y_

where d_x_ = IC_50_ of compound X in combination, D_X_ = IC_50_ of compound X alone, d_y_ = IC_50_ of compound Y in combination and D_Y_ = IC_50_ of compound Y alone.

### 3.5. Apoptosis Analysis by Annexin V FITC Binding Assay

HT-29 (1 × 10^6^) and SW837 (5 × 10^5^) cells were seeded in 60 mm plates and incubated for 24 h. HT-29 cells were treated with a mixture of 105 µg/mL of 6G and 67 µg/mL of γ-T3, while SW837 cells were treated with 70 µg/mL of 6G and 20 µg/mL of γ-T3. Cells were then incubated for 24 h and stained with 5 µL of annexin V FIT C and 5 µL of propium iodide and role of apoptosis assessed using flow cytometry.

### 3.6. Active Caspase-3 Assay

HT-29 (1 × 10^6^) and SW837 (5 × 10^5^) cells were seeded in 60 mm dishes and incubated for 24 h. HT-29 cells were treated with a mixture of 105 µg/mL of 6G and 67 µg/mL of γ-T3 while SW837 cells were treated with 70 µg/mL of 6G and 20 µg/mL of γ-T3. Cells then were incubated for 24 h and stained with FITC rabbit anti-active-caspase 3 and assessed using a flow cytometer.

### 3.7. Statistical Analysis

Results are expressed as mean ± SD. All experiments were done in triplicates and repeated at in at least three independent experiments. Statistical analysis of the results was carried out using SPSS version 20.0 and significance was set at *p* < 0.05.

## 4. Conclusions

In summary, our results suggested that combined treatment of 6G and γ-T3 shows potent anti-proliferative properties by inducing apoptosis in human colorectal cancer cells and have potential in cancer chemoprevention. Although the mechanisms of action of the individual compounds 6G and γ-T3 may be known, the underlying mechanism for the combined treatment needs further investigation.
